# Enteropathogenic *E. coli* shows delayed attachment and host response in human jejunum organoid‐derived monolayers compared to HeLa cells

**DOI:** 10.1002/1873-3468.70182

**Published:** 2025-10-13

**Authors:** Mastura Neyazi, Pilar Samperio Ventayol, Natalie Burkard, Nicolas Schlegel, Carmen Aguilar, Sina Bartfeld

**Affiliations:** ^1^ Research Centre for Infectious Diseases, Institute for Molecular Infection Biology Julius Maximilians Universität Würzburg Germany; ^2^ Institute of Biotechnology Technische Universität Berlin Germany; ^3^ Si‐M/‘Der Simulierte Mensch’ Technische Universität Berlin and Charité–Universitätsmedizin Berlin Germany; ^4^ Department of General, Visceral, Transplant, Vascular and Pediatric Surgery University Hospital Würzburg Germany

**Keywords:** EPEC infection, host immune response, human gastrointestinal organoids, organoid‐derived monolayers

## Abstract

Enteropathogenic *E. coli* (EPEC) attaches to host intestinal epithelial cells, resulting in severe illness and diarrhoea. Different cell models have been used to study EPEC infection, but a direct comparison of infection of cell lines and primary cells is lacking. In this study, we compare EPEC infection in primary epithelial cells with HeLa cells. Jejunal organoid‐derived cells contain differentiated intestinal epithelial cells and form a tight monolayer. Upon infection, they retain integrity over longer time periods than HeLa cells. Attachment of EPEC to host cells and innate immune response is strongly delayed in organoid‐derived monolayers. These results indicate that host cell factors determine the outcome of infection. Future identification of these host factors will aid the development of new therapeutics.

## Graphical abstract

Enteropathogenic *E. coli* (EPEC) infects the human intestinal epithelium, resulting in severe illness and diarrhoea. In this study, we compared the infection of cancer‐derived cell lines with human organoid‐derived models of the small intestine. We observed a delayed in attachment, inflammation and cell death on primary cells, indicating that host factors determine the outcome of infection.

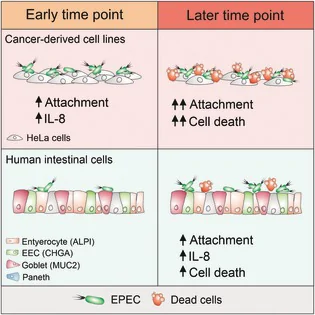

## Abbreviations

**ACE2**, angiotensin‐converting enzyme 2

**ALPI**, alkaline phosphatase 1

**CFU**, colony‐forming unit

**CHGA**, chromogranin A

**EECs**, enteroendocrine cells

**EGF**, epithelial growth factor

**EPEC**, enteropathogenic *E. coli*

**FGF**, fibroblast growth factor

**GAPDH**, glyceraldehyde‐3‐phosphate dehydrogenase

**GFP**, Green fluorescent protein

**IGF1**, insulin‐like growth factor

**IL**, interleukin

**IPTG**, isopropyl β‐D‐1‐thiogalactopyranoside

**MOI**, multiplicity of infection

**MUC2**, Mucin 2

**NF‐kappaB**, nuclear factor‐kappaB

**PI**, propidium iodide

**T3SS**, type 3 secretion system

**TEER**, transepithelial electrical resistance

**TGFβi**, transforming growth factor‐beta inhibitor

**WT**, wild‐type

**ZO‐1**, Zonula Occludens‐1

Contrary to the commensal *Escherichia coli* (*E. coli*) strains, pathogenic *E. coli* strains such as EPEC have evolved to attach to host epithelial cells and to translocate effector proteins into the host cell via the bacterial type 3 secretion system (T3SS). Secreted proteins allow further attachment and actin rearrangement leading to the formation of actin structures, termed ‘pedestals’, beneath the bacteria and the characteristic histopathologic lesion on the intestinal cells, known as the attaching and effacing (A/E) lesions [1, 2]. Attachment leads to posttranscriptional regulation in the bacteria [3].

On the host side, the epithelial lining of the gastrointestinal tract functions as a physical and immunological barrier between the microbes of the gut and the body. The intestinal cells are in constant interplay between the content of the intestine and the immune environment to maintain homeostasis and mount an immune response upon infection. The innate immune response of the host cell initiates the NF‐kappaB signalling pathway that results in pro‐inflammatory cytokine production upon microbial exposure. However, bacterial translocated proteins also inhibit the host innate immune response by blocking the NF‐kappaB pathway [4, 5].

Classically, EPEC infection is studied using cell lines including HeLa, Hep‐2, Caco‐2 and T84 [4, 5, 6, 7] which are derived from cancerous cells including cervical cancer and colorectal adenocarcinoma, respectively, as well as intestinal biopsy cultures [2]. These cell lines not only lack the heterogeneity of the intestinal epithelium and segment specificity, but they also contain many genetic mutations that might influence bacterial infection studies. Mouse models are available, but since the mouse is not a natural host, there are limitations as well [8, 9]. Therefore, additional experimental models, such as primary cells, are needed to investigate EPEC infection.

In the past decade, patient‐derived organoids have been a widely used technology [10]. Since organoids retain the characteristics of the native tissue from which they are derived, they reveal key aspects of intestinal epithelial physiology and stem cell differentiation dynamics [11, 12]. Further, organoids, their derived monolayers and 3D bioengineered models have been used to study gastrointestinal infection [13, 14, 15, 16, 17, 18]. Because these models retain cellular heterogeneity and/or cells that can be driven into specific lineages by directed differentiation, they have been particularly useful to understand human‐specific pathogens and their organ or cell tropism [19, 20, 21].

Early work already used ileal and rectal organoid‐derived monolayers for infection with *E. coli* and revealed that there may be different adherence patterns for various diarrheagenic *E. coli* that are different from the adherence patterns to the classical HeLa cell monolayer [14]. However, a more detailed comparison of HeLa cells with intestinal organoid‐derived primary cell infection dynamics; adherence and immune response to EPEC infection is lacking. Understanding the differences in the infections of HeLa and intestinal cells may provide novel insights into host factors determining the outcome of infection, paving the way for intervention measures for controlling re‐emerging diseases caused by EPEC.

Here, we provide a direct comparison of EPEC adherence and immune response in HeLa cells and jejunal organoid‐derived monolayers for the first 6 h of infection. Results highlight lower bacterial attachment, less epithelial cell death and a delayed inflammatory response in jejunal monolayers compared to HeLa cells. This suggests higher resilience to infection in the primary cells than in HeLa cells. This points to differences in the two cell models that can reveal host factors important for bacterial attachment in the future.

## Material and methods

### Cell culture

HeLa‐229 cells (CVCL_1276, obtained from Ana Eulalio laboratory, Institute for Molecular Infection Biology, Julius Maximilians Universität Würzburg) were cultured in RPMI 1640 media supplemented with 10% FBS. Caco‐2 cells (CVCL_0025, obtained from Cynthia Sharma laboratory, Institute für Molecular Infection Biology, Julius Maximilian Universität Würzburg) were cultured in DMEM (Corning 10‐013) supplemented with 10% FBS and 1% amino acids (Biowest X0557). 50 000–60 000 cells were seeded on 48‐well plates (833 923, Sarstedt) 1–2 days prior to infection. Cells were used at 70–100% confluency. Subsequent infection and analysis were the same as in jejunal monolayers.

### Organoid culture and 2D monolayer seeding

Gastrointestinal tissue material was obtained from 5 donors (age range 34–53) (Table S1), that underwent surgical resection at the University Hospital of Würzburg. The study was approved by the ethical committee of the University of Würzburg (Approval 37/16), informed consent was obtained from all donors, and the study methodologies conform to the standards set by the Declaration of Helsinki. Organoids were generated following previously published protocols [22].

Organoids were cultured and expanded in organoid expansion media. For propagation, organoids were split every 10–12 days at a ratio of 1:4 using mechanical splitting. Culture medium was changed every 2–3 days. Propagation culture medium contained: 30% base media: Advance Dulbecco's modified Eagle medium (DMEM)/F12 (12 634 028; Thermo Fischer Scientific, Dreieich, Germany) supplemented with 10 mm HEPES (15 630 056, Thermo Fischer Scientific), 1x GlutaMAX (35050‐038; Thermo Fischer Scientific) (AD++), 1×B27 (12 587 010; Thermo Fischer Scientific), 1 mm N‐Acetylcysteine (A9165‐25G; Sigma‐Aldrich, St. Louis, MO, USA), 10% Noggin conditioned medium, 10% R‐spondin 1 conditioned medium, 50% Wnt3a conditioned medium (for production protocol, see [23]), 10 nm Gastrin (3006, Tocris, Bristol, UK), 0.5 μm TGFβi (A‐83‐01, Tocris), 100 ng·mL^−1^ IGF‐1 (590 904, BioLegend, San Diego, CA, USA), 50 ng·mL^−1^ FGF‐basic (C100‐18B, PeproTech, Cranbury, NJ, USA), 50 ng·mL^−1^ EGF (AF‐100‐15, PeproTech, Cranbury, NJ, USA), and 100 ng·mL^−1^ Primocin (Ant‐pm‐1, Invivogen, San Diego, CA, USA). After seeding, 10 μm RHOKi (Y‐27632, Sigma‐Aldrich) was added.

Monolayers were generated from organoids 5–6 days after seeding. Organoids were collected in AD++ and centrifuged at 450 ***g*** for 5 min, resuspended in TrypLE Express (12 605 028, Gibco, Waltham, MA, USA), incubated at 37 °C for 10 min and mechanically disrupted. Single cell suspension was washed with AD++ and centrifuged at 450 ***g*** for 5 min. 60 000–80 000 single cells were seeded in 48‐well plate (833 923, Sarstedt, Nümbrecht, Germany), using the maintenance media supplemented with RHOKi (10 μm, Y‐27632, Sigma‐Aldrich). 48‐well plates and μ‐Slide 8 Wells (80 826, Ibidi, Gräfelfing, Germany) were coated with collagen 1, 15 μg·cm^−2^ in filtered 0.02% Acetic acid in PBS. Following day, maintenance media were replaced with media free of Primocin. Monolayers were grown for 7–10 days to reach 70–100% confluency before further analysis and EPEC infection.

### Transepithelial electrical resistance

TEER measurements were performed as described before [24]. To evaluate TEER, cells were seeded on 8‐well PET slides with 40 electrodes per well (8W10E+, Applied Biophysics, Troy, NY, USA). Measurements were done at 400 Hz, starting at day 5 postseeding using ECIS® 8‐well transfilter Array connected to ECIS^®^ Z‐Theta (Applied Biophysics), and continued until day 12; media were exchanged every 2–3 days.

### Infection of cells

EPEC strains were gifted by Ilan Rosenshine, Institute for Medical Research, Hebrew University of Jerusalem (Table S2). Protocol published previously [4] was followed for EPEC infection. In brief, EPEC wt and Δ*nleBCDE* mutant carrying a plasmid expressing GFP were cultured overnight in LB‐medium (Lennox, 6669.1, Roth, Karlsruhe, Germany) supplemented with 0.1 mg·mL^−1^ Ampicillin, without shaking. The following day, the bacteria culture was diluted (1:100) in DMEM media (31 966 047, Thermo Fisher Scientific) and cultured for 3 h at 37 °C, without shaking. 10 μm IPTG (2316.3, Roth) was supplemented in the DMEM culture to activate GFP expression. For jejunal monolayers, 60 000–80 000 single cells were seeded in a 48‐well plate. Monolayers were used at day 7–10 after seeding, based on the confluency of the wells. Confluencies of 70–100% were used for infection. For HeLa, 50 000 single cells were seeded in a 48‐well plate. Monolayers were used at day 1–2 after seeding, based on the confluency of the wells. Confluencies of 70–100% were used for infection. At 100% confluent monolayers, we quantified 70 000 HeLa cells per well and 110 000 jejunal cells per well. Calculations of the MOI for the inoculation were based on individual confluencies and deduced cell numbers. Cells were washed 3 times with PBS after infection. Cells were subjected to further analysis.

### Live cell imaging

Jejunum (pooled cells from Pat. #44, 50, 84) and HeLa cells were seeded on μ‐Slide 8 Wells (80 826, Ibidi), numbers described above, and grown to full confluency, 2 days for HeLa cells and 8 days for jejunal monolayers. Fresh media with 50 ng·mL^−1^ of propidium iodide (51‐6621E, BD Pharmingen, San Diego, CA, USA) was added to the cells before infection and incubated at 37 °C for 15 min. They were then infected with GFP EPEC wt at MOI 1 and imaged for 6 h. Imaging started at 0 hpi using a Leica DMi8 inverted microscope and a Leica MDI6000 B fluorescent live cell imaging system, using 488 nm and 561 nm laser frequencies for excitation. Images were taken every 10 min, using a 20× dry objective. All images were analysed using imagej software.

### Statistical analysis

All statistical analysis was performed using Prism 9 (GraphPad software). Results are presented as mean with standard error of means (SEM). For comparisons between two groups, the nonparametric Mann–Whitney test was used. Significance values are shown in each graph.

## Results

### Cells derived from human jejunal organoids grow into tight monolayers

To allow a direct comparison of EPEC attachment between HeLa and organoid‐derived cells, we first aimed to generate an organoid‐derived monolayer. For this, well‐expanded organoids were enzymatically dissociated and seeded on collagen 1 precoated plates (Fig. 1A). The cells proliferated and formed confluent monolayers in 8 days (Fig. 1B). The timepoint of confluency depended on the number of seeded cells (data not shown). The apical side of the cells faced the lumen of the well as indicated by ZO‐1 and F‐actin staining (Fig. 1C,D) and retain columnar morphology as observed with Z sections (Fig. 1D). The cells formed a tight monolayer with high transepithelial electrical resistance (TEER) (Fig. 1E).

**Fig. 1:**
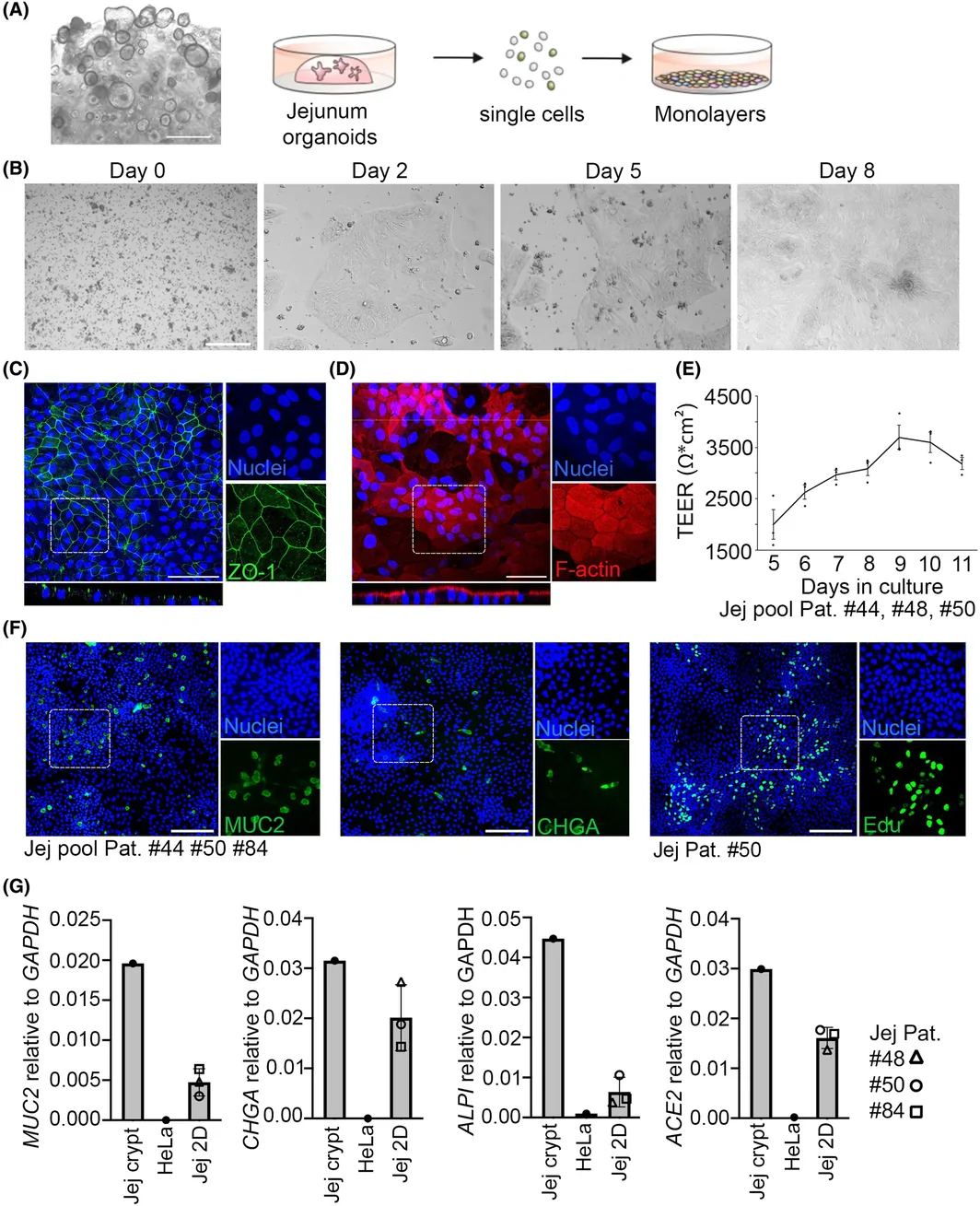
Cells derived from human jejunum organoids grow into tight monolayers. (A) Organoids and scheme of experiment. Scale bar – 400 μm. (B) Brightfield images of jejunal monolayer at day 0, 2, 5 and 8 postseeding. Scale bar – 200 μm. (C) Images of immunofluorescence staining for tight junction protein, Zonula Occludens‐1 (ZO‐1, green) and staining for nuclei using Hoechst 33342 (blue), scale bar – 100 μm. (D) Staining of F‐actin with phalloidin (red) and nuclei with Hoechst 33342 (blue). Scale bar – 50 μm. (E) Transepithelial electrical resistance (TEER) analysis of jejunal monolayers from day 5 to day 11 postseeding, results show 3 independent experiments, each represented with a dot, and each performed with a pool of cells from 3 patients (Pat#44, #48 and #50). Bars represent mean with standard error of the mean (SEM) of 3 individual experiments. (F) Images of monolayers immunostained for goblet cell marker mucin 2 (MUC2), enteroendocrine cell marker chromogranin A (CHGA) or stained using incorporation of EdU indicating proliferation (green) and stained with Hoechst 33342 for nuclei (blue). Scale bar – 100 μm. (G) Jejunal monolayers were cultured for 7–8 days until 100% confluent. Cells were harvested, RNA was prepared and expression of *MUC2*, *CHGA*, Alkaline phosphatase 1 (*ALPI*) and angiotensin converting enzyme 2 (*ACE2*) genes were quantified by RT‐qPCR analysis. Results were normalised to Glyceraldehyde‐3‐phosphatase dehydrogenase (*GAPDH*). As control, material from jejunal crypts isolation (Patient 50) before generation of organoids was used as described before [22]. Bars represent mean with SEM of 3 individual patients.

The presence of proliferating cells was confirmed by EdU staining (Fig. 1F). Differentiation was evident by immunofluorescence staining and RT‐qPCR for mucin 2 (*MUC2*) for goblet cells, chromogranin A (*CHGA*) for enteroendocrine cells (EECs) and alkaline phosphatase (*ALPI*) and angiotensin converting enzyme 2 (*ACE2*) for enterocytes [13] (Fig. 1F,G) (Table 1). On average, the monolayers consisted of 4% MUC2+ goblet cells and 1.2% CHGA+ EECs (data not shown). For the RT‐qPCR, HeLa cells were used as a negative control and freshly isolated human epithelial cells as a positive control. Together, these data indicated that cells from human jejunal organoids could form a tight monolayer comprising proliferative cells and differentiated cells.

**Table 1:** Primer list used in this study.

Gene	Forward	Reverse
*GAPDH*	5′ CTCTCTGCTCCTCCTGTTCGAC	5′ TGAGCGATGTGGCTCGGCT
*ALPI*	5′ CAGATGCTGACATGCCTGCC	5′ TTTCGTGTCTCCGGGCTCA
*MUC2*	5′ CGTCCGTCTCCAACATCACC	5′ CACCCTGGTCTCATTGCGAG
*CHGA*	5′ TCCGAGGAGATGAACGGATCC	5′ GGATGATGGCTCTTCCACCG
*ACE2*	5′ TCAGAACCCTGGACCCTAGC	5′ GTTCTGGTCTTTCAGCCAGGT
*IL‐8*	5′ ACACTGCGCCAACACAGAAAT	5′ ATTGCATCTGGCAACCCTACA

### EPEC adherence is lower in intestinal primary cells than in HeLa cells

To compare EPEC infection in the classical HeLa model and jejunal organoid‐derived monolayers, we infected both models in parallel. To assess adherence, we used GFP‐expressing variants of the bacteria at multiplicity of infection (MOI) 1, 10 and 100. Fluorescence analysis showed less attachment of the bacteria to the organoid‐derived monolayers compared to HeLa cells (Fig. 2A). These observations were quantified using flow cytometry at 3 hpi. Again, jejunal monolayers showed less attachment of EPEC (40% of cells positive with EPEC) compared with HeLa cells (90% of cells positive with EPEC) (Fig. 2B). The same trend was observed with colony‐forming unit (CFU) counts after plating of the bacteria from the monolayers at 3 hpi (Fig. 2B). Time course analysis showed that this trend was consistent over time. In an infection with MOI 10, jejunal monolayers showed less cells positive with EPEC than in HeLa cells: A maximum of 40% of jejunal cells were positive with EPEC compared to 70% of HeLa cells positive with EPEC. This outcome was also reflected in the CFU counts at 1, 2, 3 and 4 hpi (Fig. 2C). EPEC growth in the culture medium from HeLa cells and jejunal monolayers showed little difference over 12 h, ruling out a general influence from the host cell media component (Fig. S1). This indicated that EPEC attachment was reduced or delayed in jejunal monolayers compared to HeLa cells.

**Fig. 2:**
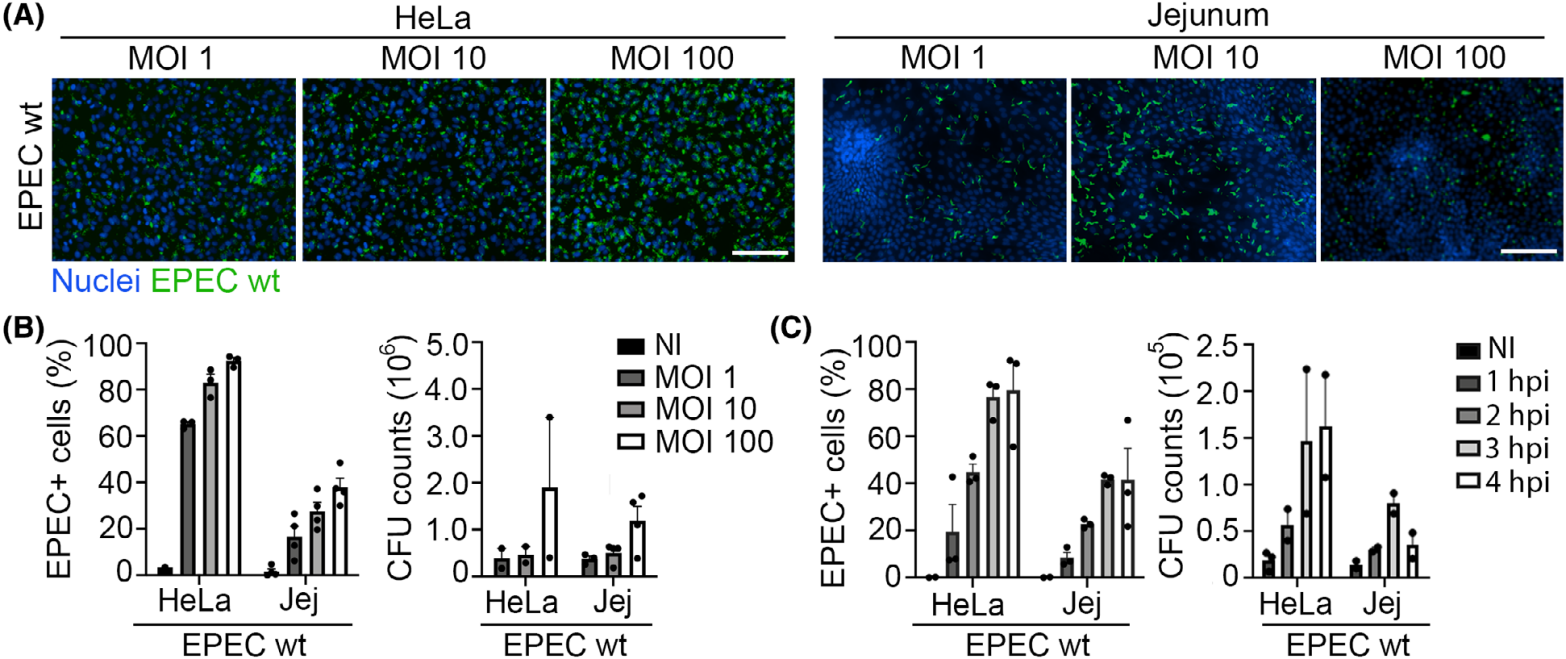
EPEC attachment is lower in jejunal monolayers compared to HeLa cells. (A) HeLa cells and jejunal monolayers were infected with GFP EPEC wt MOI 1, 10 or 100 for 3 h. Nuclei were stained with Hoechst 33342. Scale bar – 100 μm. (B) Quantification of adhered bacteria to HeLa cells and jejunal monolayers with flow cytometry and colony forming unit (CFU) counts. (C) Quantification of adhered bacteria to HeLa cells and jejunal monolayers with flow cytometry and CFU at indicated timepoints (hours postinfection) and MOI 10. Cells were infected at 100% confluency. Bars in B and C represent mean with standard error of the mean (SEM) of organoids from 2 to 4 donors, each dot represents results from organoids of one donor.

### Intestinal primary cells retain monolayer integrity with EPEC infection over a period of 6 h

The low EPEC adherence in the small intestinal organoid‐derived monolayers prompted us to ask, whether the host cells can retain this adherence difference over a longer period of time.

For this, bacteria were added at an MOI of 10 to 100% confluent monolayers of jejunal cells or HeLa cells. Both models displayed characteristic actin filament rearrangement on the apical side of the host cells beneath the bacteria (Fig. 3A,B), one of the hallmarks of EPEC infection [7]. Over 6 h of infection with EPEC, we observed that HeLa cells were detaching and dying with a higher number of PI‐positive cells (red), whereas jejunal monolayers were intact with only a few cells budding off from the monolayers, which were also PI positive (Fig. 3C; Fig. S2A). Quantifying the percentage of dying cells with PI staining at 3, 4 and 6 hpi in HeLa cells and jejunal monolayers showed significantly higher percentages of HeLa cells PI positive at 4 hpi and 6 hpi, 50% and 90% respectively, compared to jejunal monolayers, where 40% of cells were PI positive at 6 hpi (Fig. 3D; Fig. S2B). To exclude that this difference was rooted in the specificity of the HeLa cell line, we also included a commonly used intestinal cell line, Caco‐2. Caco‐2 cells were infected with different MOIs and showed similar attachment and PI‐positive cell numbers as HeLa cells (Fig. S2C,D). This suggested that small intestinal organoid‐derived monolayers were more resilient in experimental infection compared to HeLa cells over 6 h of infection.

**Fig. 3:**
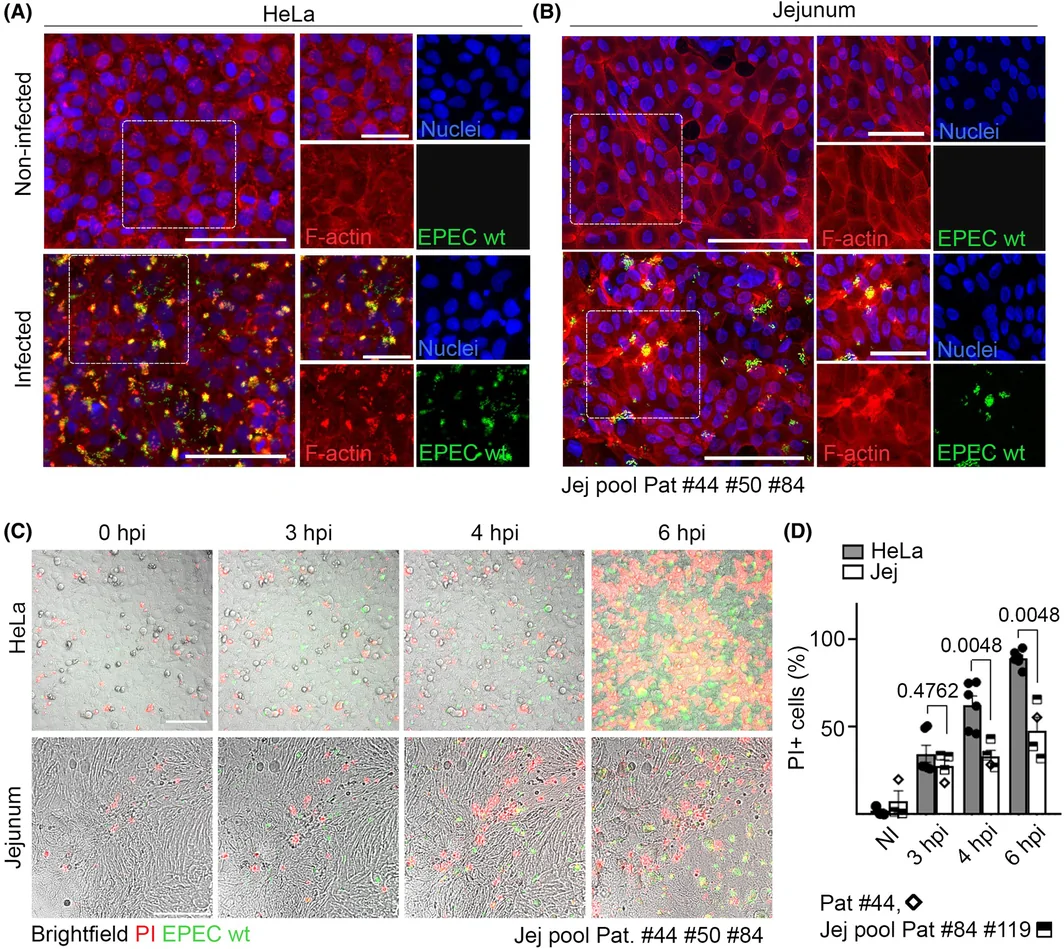
Intestinal primary cells retain prolonged monolayer integrity with EPEC infection compared to HeLa cells. (A) Representative images of EPEC attachment in HeLa cells 3 hpi with GFP EPEC wt at Multiplicity of infection (MOI) 10 (green). Cells were counterstained with phalloidin for F‐Actin (red) and Hoechst 33342 for nuclei (blue). (B) Representative images of EPEC attachment in jejunal monolayer 4 hpi with GFP EPEC wt at MOI 10 (green). Cells were counterstained with phalloidin for F‐Actin (red) and Hoechst 33342 for nuclei (blue). (C) Brightfield images of HeLa cells and jejunal monolayers incubated with GFP EPEC wt MOI 1 at time points 0, 3, 4 and 6 hpi, Cells were incubated for 15 min with propidium iodide (PI) staining (red) before adding the bacteria. (D) Quantification of PI+ HeLa cells and jejunal monolayers non infected (NI) and at 3, 4 and 6 hpi with GFP EPEC wt MOI 1. PI was added 15 min before cytometric analysis. Data represent mean with standard error of the mean (SEM) of one experiment with Pat#44 and 2 independent experiments with a pool of cells from 2 patients Pat#84/119. Each experiment was performed in parallel with HeLa in 2 technical replicates. The 3 separate experiments are shown in Fig. S2B. Statistical analysis was performed using nonparametric Mann–Whitney test using GraphPad Prism 9. Scale bar in (A, B) – 100 μm, 50 μm (inserts) and in (C) – 100 μm.

### NF‐kappaB host response is delayed in jejunal monolayers compared to HeLa cells

To better define reduced or delayed attachment, we analysed the inflammatory response of the host cells to infection. Attached EPEC translocate a range of effector proteins into the host cells. Of these, several can also suppress the activation of the NF‐kappaB response. To assess the NF‐kappaB response, here we used a previously published strain that has been constructed to lack all effectors suppressing NF‐kappaB, EPEC Δ*nleBCDE* [4]. To first ensure that the mutants do not show altered attachment, we directly compared the attachment of the wild‐type (wt) and mutant (Δ*nleBCDE*) strains to HeLa and jejunal monolayers. Data showed comparable attachment as measured in fluorescent analysis, cytometric quantification and CFU counts (Fig. S3A–C). Thus, the Δ*nleBCDE* mutation does not impact attachment. To then assess the immune response of the primary epithelial cells to EPEC, we analysed the NF‐kappaB subunit p65 translocation to the nuclei in HeLa cells and jejunal monolayers (Fig. 4A). Quantification of nuclear p65+ cells in HeLa and jejunal monolayers showed that at 3 hpi, 50% of the HeLa cell nuclei are positive for p65, whereas in jejunal monolayers, 18% of the nuclei were p65+ (Fig. 4B). At 4 hpi, both cultures showed about 45–50% of p65+ nuclei (Fig. 4B; Fig. S3D,E). Similarly, at the early timepoint, 3 h, *IL‐8* expression was significantly higher in HeLa cells than in jejunal monolayers when infected with EPEC Δ*nleBCDE* (Fig. 4C). However, the analysis at 6 h showed that *IL‐8* expression was delayed but not diminished in jejunal monolayers compared to HeLa cells (Fig. 4C; Fig. S3F,G). This suggested that the jejunal monolayers were not generally protected from attachment and NF‐kappaB activation, but rather that there was a delay compared to HeLa cells.

**Fig. 4:**
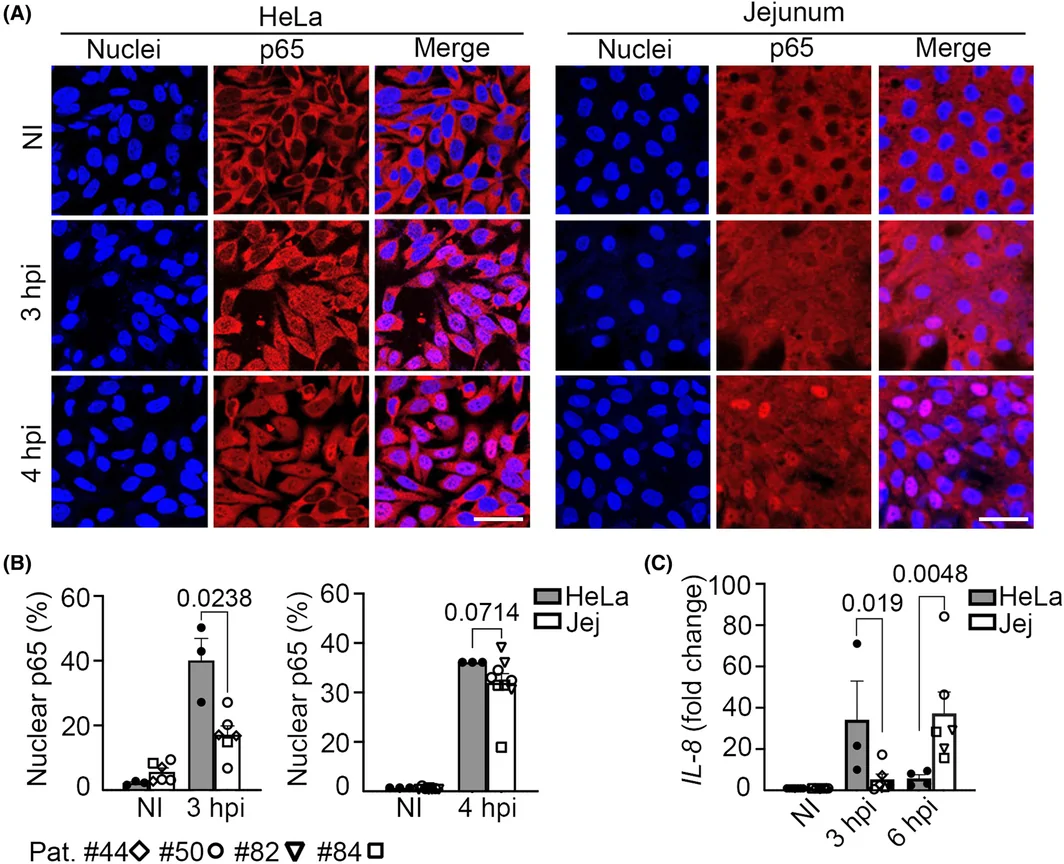
NF‐kappaB host response is delayed in jejunal monolayers compared to HeLa cells. (A) Immunofluorescence staining of NF‐kappaB subunit p65 in HeLa cells and in jejunal monolayers in response to EPEC Δ*nleBCDE* at 3 and 4 hpi (scale bar – 50 μm). (B) Quantification of nuclear p65‐positive cells at 3 and 4 hpi. Images and figures represent 4 donors and 3 independent experiments which are shown separately in Fig. S3D,E. Each data point represent average of 9 images per well for each condition. (C) *IL‐8* gene expression analysis in HeLa cells and jejunal monolayers infected with EPEC Δ*nleBCDE* at 3 and 6 hpi. Experiment representative of 3 independent experiments which are shown separately in the Fig. S3F,G. Data represent mean with standard error of the mean (SEM) of at least 3 independent experiments and 4 individual donors. Statistical analysis was performed using nonparametric Mann–Whitney test using GraphPad Prism 9.

Together, the results suggested that primary intestinal cells were better guarded to protect from EPEC infection than HeLa cells. EPEC adherence to primary epithelial cells was reduced compared to HeLa cells. A delayed inflammatory immune response is likely a consequence of the delayed adherence.

## Discussion

For EPEC, adherence to host cells and dampening immune response to the bacteria is particularly important not only in successful colonisation of the intestine but also in establishing long‐term infection. Until recent years, cancerous cell lines such as HeLa cells have been the primary *in vitro* model for studying the pathophysiology of EPEC [4, 5, 6, 7]. Here we show a direct comparison of EPEC adherence and immune response in HeLa cells and jejunal organoid‐derived monolayers for the first 6 h of infection. Results highlight lower bacterial attachment, less epithelial cell death and a delayed inflammatory response in jejunal monolayers compared to HeLa cells.

In severe infections *in vivo*, the epithelium is disrupted, leading to extensive atrophy and thinning of the mucus lining [25]. The initial adherence of EPEC to the epithelium is thought to be crucial in this pathogenesis because of the A/E lesions [26]. In this light, the high resilience of the primary epithelial cells observed here may be initially surprising, but it is mirrored by the findings from early experimental infections *in vivo*. In piglets, the intestinal epithelial cells and the brush border were found to remain intact after 6 h of infection with EPEC, even with an increase in the number of luminal bacteria [27]. This was further reported in experimental infection of human duodenal biopsies, where samples retained morphological preservation even with EPEC colony size increasing with incubation time and eventually colonising the entire mucosa at 12‐h postinfection [28, 29]. Interestingly, the pathological characteristics for EPEC were observed after 24 and 48 h of infection [25, 28]. Thus, while the majority of HeLa cells undergo cell death within 6 h of infection, the primary jejunal monolayers remain largely intact during this period, better reflecting the preserved epithelial integrity observed in classical *in vivo* studies.

The presence of differentiated cells in organoid‐derived monolayers, however, can influence the outcome of infection in several ways. First, presence of a putative specific target cell may influence the infection. Previous research has shown EPEC binding to terminally differentiated cells, enterocytes, the major absorptive cell population [2]. Second, general cellular morphology and the expression of tight junctions can influence infection. EPEC effector proteins have been shown to target the tight junction complex which acts as a barrier and regulates the passage of water and solutes, that result in microtubule disruption [30, 31]. It seems possible that the differences in junctional proteins between HeLa and jejunal cells may contribute to the different outcomes of infection. Third, mucus‐producing goblet cells and production of antimicrobial proteins could contribute to the presence of a protective mucus barrier. These hypotheses will be further investigated in the future.

We chose to work with 2D organoid‐derived monolayers. Like all models, this system has limitations: it lacks the 3D architecture and cellular complexity of native tissue. However, it allows apical access, measurement of barrier integrity and standardised infection protocols without the need for microinjection. In comparison, 3D organoids can capture crypt–villus or gland–pit structures more faithfully, but restrict apical exposure. Importantly, a study in human gastric organoids could show that in both models, 2D organoid‐derived monolayers and 3D organoids are capable of producing the same cell types, which is likely rooted in the capacity of the adult stem cells [18]. Several recent reviews have highlighted the advantages and disadvantages of different organoid‐centred model systems [19, 20, 32, 33]. Ultimately, no single model fully reflects the complexity of the *in vivo* epithelium, and 2D monolayers offer a tractable compromise for dissecting host–pathogen interactions at the epithelial surface under defined conditions.

Understanding the differences in the infections of HeLa and primary intestinal cells may provide novel insights into host factors determining the outcome of infection, paving the way for intervention measures for controlling re‐emerging diseases caused by EPEC. Beyond their relevance for understanding host–pathogen interactions, organoid‐derived intestinal models offer a promising platform for therapeutic development. Their ability to recapitulate the cellular diversity, polarity and barrier function of the native human epithelium makes them well‐suited for high‐content drug screening and evaluation of anti‐infective strategies. For instance, these models could be used to assess the efficacy of small molecules or biologicals targeting bacterial adhesion, T3SS effector function or host inflammatory responses. Additionally, they could potentially improve the predictive power of preclinical testing for enteric pathogens like EPEC. This may be particularly valuable in identifying compounds that stabilise epithelial integrity or modulate host responses without broadly suppressing inflammation.

## Author contributions

MN, investigation, methodology, visualisation, writing—original draft, writing—review and editing. PSV, CA and NB, methodology, writing—review and editing. NS, funding acquisition, resources, writing—review and editing. SB, conceptualisation, funding acquisition, supervision, writing—review and editing.

## Supporting information


**Fig. S1.** EPEC grows similarly in culture media for HeLa or jejunum monolayer.
**Fig. S2.** EPEC infection over a time course of 6 h; differences between jejunum monolayer and tumour‐derived cell lines.
**Fig. S3.** EPEC attachment in HeLa cells and jejunal monolayers, comparison of EPEC wt and Δ*nleBCDE* mutant.
**Table S1.** Donor list for this study.
**Table S2.** List of bacterial strains used in this study.

## Data Availability

The data are available upon request from the corresponding author. There are restrictions on the availability of human organoid cultures due to the limitations of the consent that was given by the patients and the guidance of the ethical committee.
