# Supplementary material for: Enteropathogenic E. coli shows delayed attachment and host response in human jejunum organoid‐derived monolayers compared to HeLa cells
Source: FEBS Lett. 2025 Oct 13;600(21):3241–51. doi: 10.1002/1873-3468.70182 (PMC13745866; doi:10.1002/1873-3468.70182)
Supplement: Supplementary file 1 [file FEB2-600-3241-s001.pdf]

Supplementary material

- Supplementary figures
- Supplementary methods
- Supplementary tables

Supplementary figures

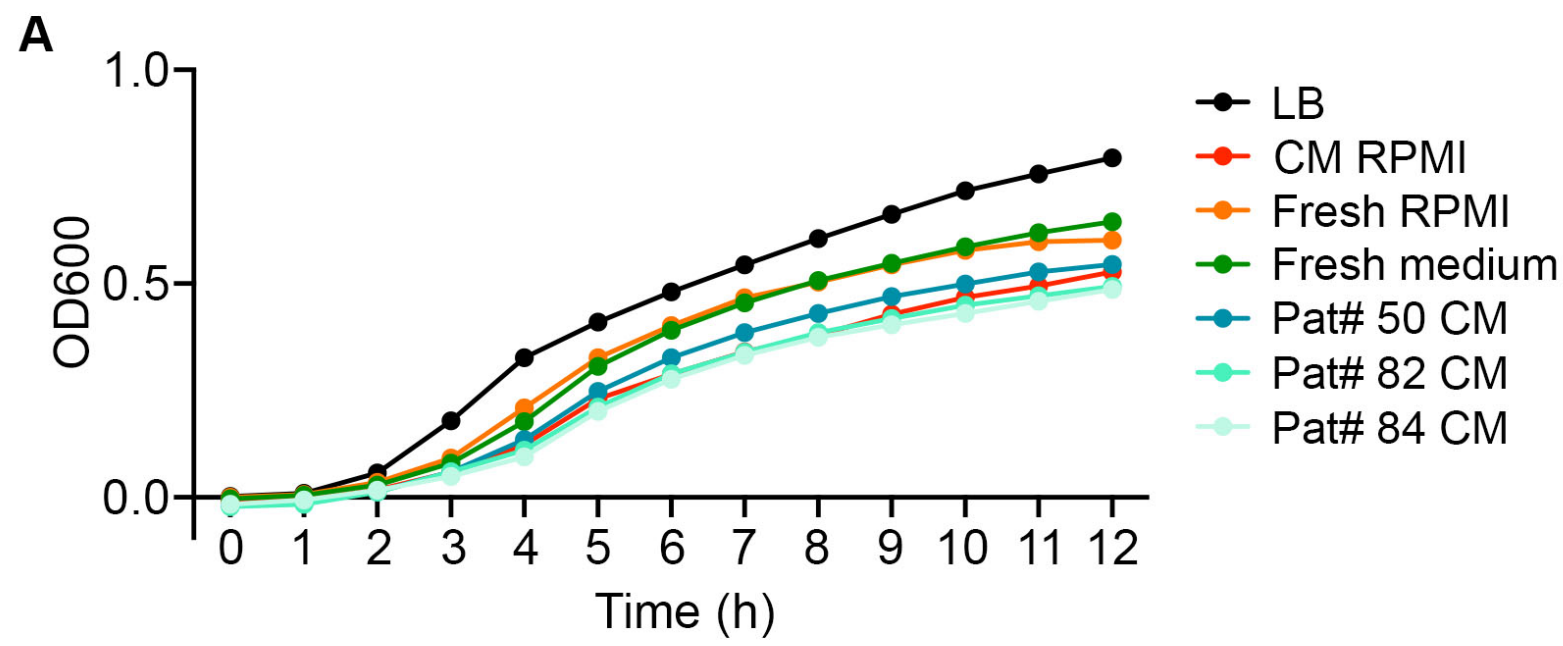

Figure S1. EPEC grows similarly in culture media for HeLa or jejunum monolayer. OD measurements of EPEC wt cultured in conditioned media from HeLa cells (CM RPMI) and conditioned jejunal monolayers from 3 patients (Pat# 50, 82, 84 CM). Supernatants were collected after 2 days of media exchange and inoculated with EPEC wt MOI 10, and measured OD for 12 hours.

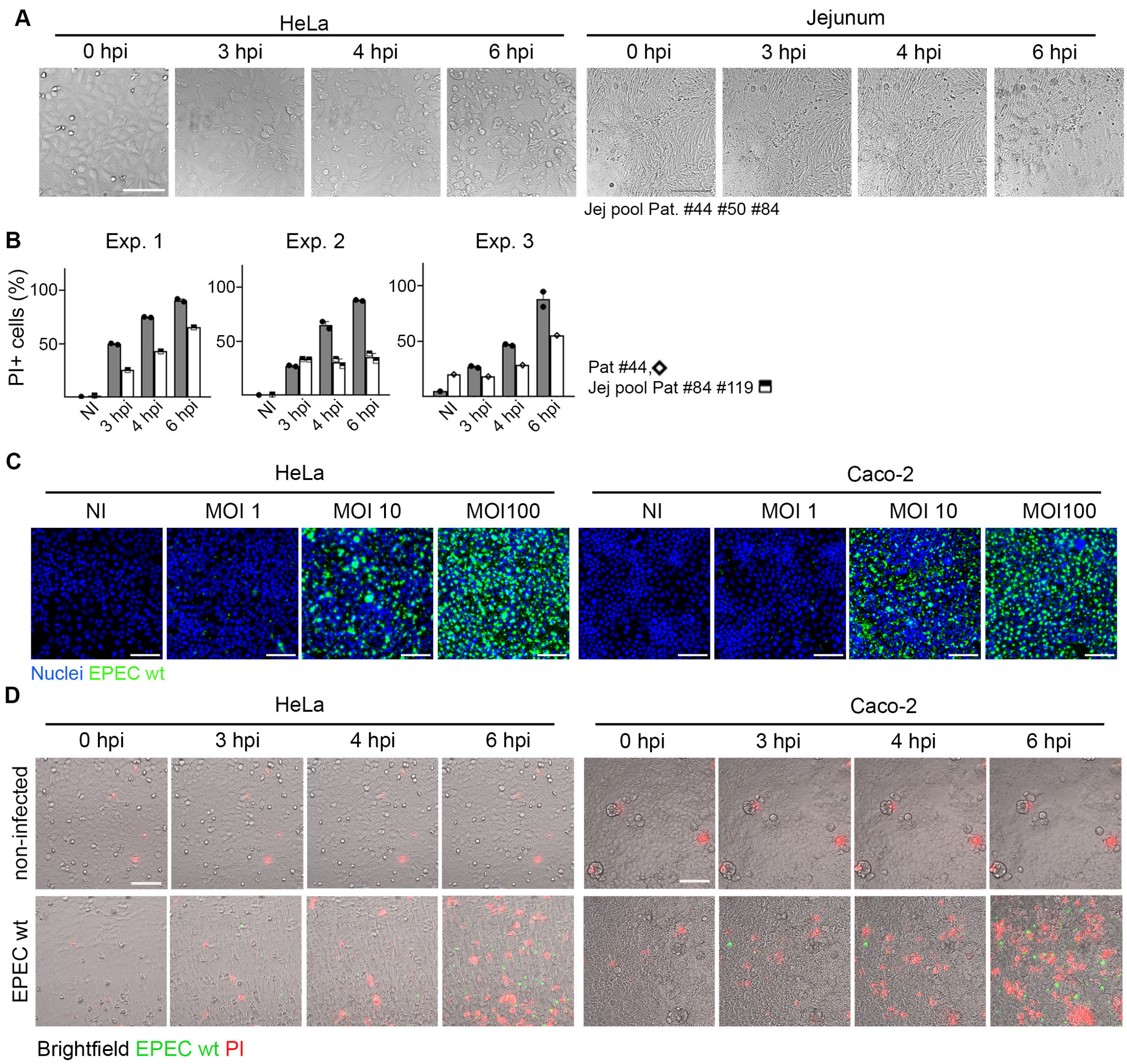

Figure S2. EPEC infection over a time course of 6 h; differences between jejunum monolayer and tumor-derived cell lines. A) Brightfield images of HeLa cells and jejunal monolayers incubated with EPEC wt at time points 0, 3, 4, and 6 hpi with MOI 1, scale bar – 100 µm. B) Quantification of PI+ HeLa cells and jejunal monolayers NI and at 3, 4 and 6 hpi post infection with EPEC wt and MOI 10 in 3 separate experiments (Exp) with individual patient replicates. This is the 3 experiments underlying Figure 3C, the data is identical. C) HeLa and Caco-2 cells were either left non-infected (MOI 0) or infected with GFP EPEC wt for 3 hours with MOI 1, 10 and 100. Nuclei were stained with DAPI. Scale bar – 100 µm. A representative experiment from 2 independent experiments is shown. D) HeLa and Caco-2 cells were infected with GFP EPEC wt at MOI 1 in presence of propidium iodide (red, PI). Live imaging was performed every 30 min for 6 h. Scale bar – 100 µm. A representative experiment from 2 independent experiments is shown.

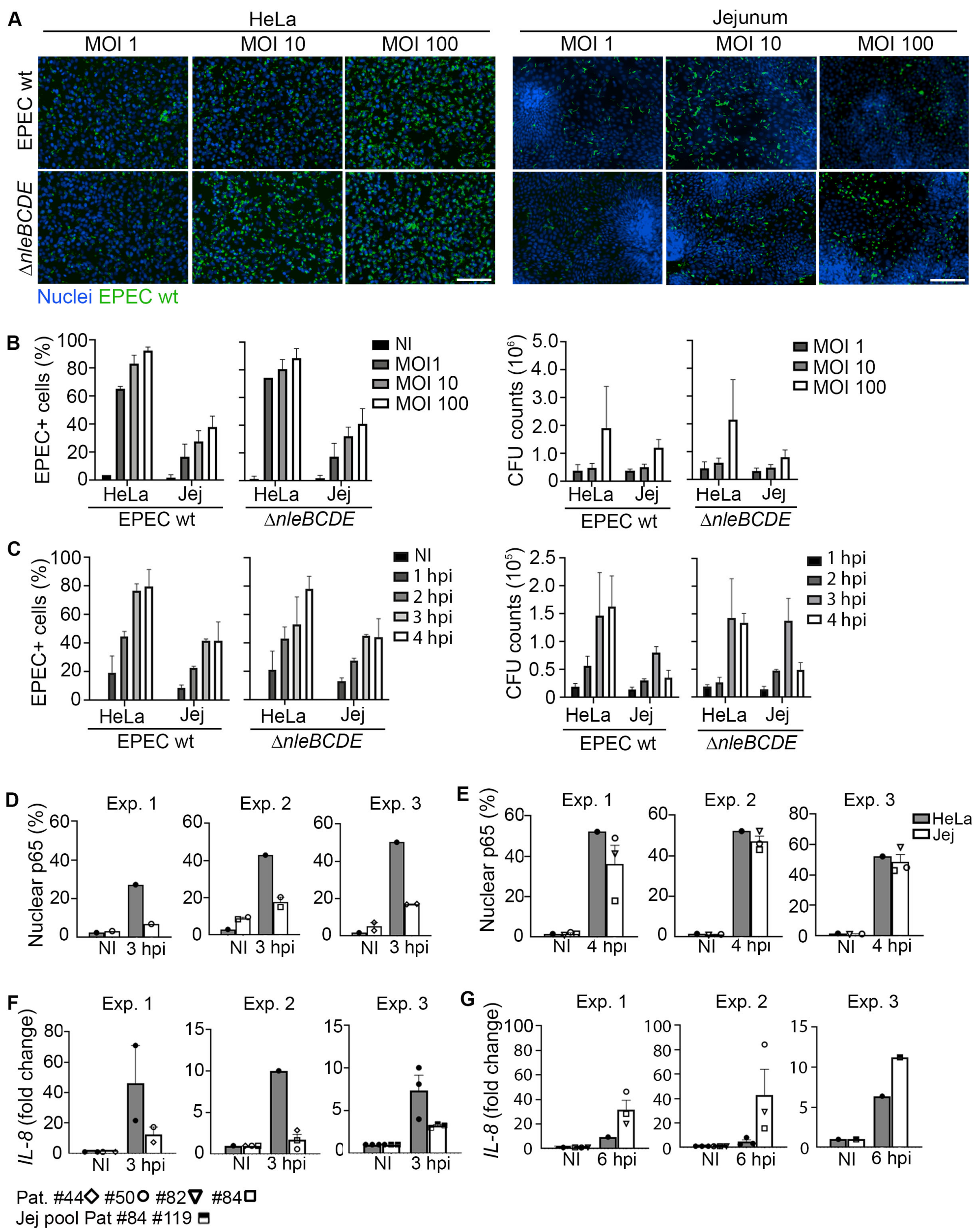

Figure S3. EPEC attachment in HeLa cells and jejunal monolayers, comparison of EPEC wt and Δ*nleBCDE* mutant. A) HeLa cells and jejunal monolayers were infected with GFP EPEC wt and Δ*nleBCDE* mutant for 3 hours with MOI 1, 10 and 100, Nuclei were stained with Hoechst, scale bar – 100 µm. B) FACS analysis and colony forming unit (CFU) counts of MOI 1, 10 and 100. Data of infections with WT is the same data as shown in Figure 2. C) FACS and CFU quantified at indicated timepoints (hours post infection). Data of infections with WT is the same data as shown in Figure 2D). D-E) Quantification of nuclear p65+ cells in non-infected (NI) and at 3 hpi (D) or 4 hpi (E) in HeLa and jejunal monolayer cells of 3 independent experiments in response to EPEC Δ*nleBCDE*. F-G) *IL-8* gene expression analysis in HeLa cells and jejunal monolayers infected with EPEC Δ*nleBCDE* at 3 hpi (F) or 6 hpi (G). D-G show identical data as Figure 4 B-C, but independent experiments individually plotted.

Supplementary material and methods

Flow cytometry and CFU analysis

After infection, cells were washed 3 times with PBS, incubated with TrypLE Express (12605028, Gibco) for 10 min, and washed twice with FACS buffer (PBS + 10% FBS). Cells were then resuspended in FACS buffer and Propidium Iodide Ready Flow™ Reagent (R37169, ThermoFisher Scientific), according to the manufacturer´s protocol. Single cells were gated by using forward scatter area versus forward scatter peak linear. GFP (EPEC wt and mutant strains) were measured in 10,000 cells. Cells were analysed using the Accuri™ C6 (BD Biosciences) and data were analysed with the Accuri™ C6 software (BD Biosciences) and FlowJo™ software package (BD Biosciences).

To quantify adhered bacteria by CFU assay, after infection cells were lysed with 0.1% Triton X-100 in PBS. Cell lysates were serially diluted, plated in LB-agar (Lennox) vegetal (0159.1, Roth) plates, and left to grow for overnight.

RNA isolation and qRT-PCR

Isolated jejunal crypts, organoids or cell monolayers were washed with PBS and harvested in RLT buffer (79216, Qiagen). Total RNA was extracted using the RNeasy Mini Kit (74106, Qiagen) according to the manufacturer´s instructions. RNA was then reverse-transcribed using hexameric random primers (48190011, Life Technologies) and M-MuLV reverse transcriptase (M0253, New England Biolabs) according to the manufacturer´s instructions. Quantitative real-time PCR analysis was performed according to the manufacturer´s instructions, in a CFX96 Touch Real-Time PCR detection system using Bio-Rad CFX Manager software (BioRad). The 10 µl PCR reaction included 1 µl of 1:5 diluted cDNA as template, 5 µl of SsoAdvanced Universal SYBR Green Supermix (172-5270, BioRad) and 1 µl of each primer (10 µM). Primers used in this study are listed in **Table 1**. The $2^{-\Delta Ct}$ method was used to calculate fold changes.

Confocal microscopy

To analyse organoid-derived monolayers, single cells were seeded on µ-Slide 8 Wells (80826, Ibidi) and expanded for 8 days. Cells were fixed with 4% paraformaldehyde for 20-30 min at RT, permeabilized with 0.3% Triton X-100 in PBS for 1 h and then blocked with 1% BSA for 1 h. Primary antibodies were diluted in blocking solution and incubated overnight at 4 °C. Primary antibodies used in this study were: anti-MUC2 was used at 1:400 (sc-15334, Santa Cruz

Biotechnology), anti-CHGA was used 1:400 (sc-28368, Santa Cruz Biotechnology), anti-ZO-1 used at 1:300 (33-910, Invitrogen) and Phalloidin was used 1:500 (A-22283, Molecular Probes). The secondary antibodies goat anti-mouse Alexa Fluor-488 (1:500, A-11001, Thermo Fisher Scientific) and goat anti-rabbit Alexa Flour-488 (1:500, 4412, Cell Signaling) were also diluted in the blocking solution and incubated at RT for 1 h. Click-iT® EdU Alexa Fluor® 488 (C10337, ThermoFisher Scientific) Imaging Kit was used according to manufacturer's instructions. Nuclei were counterstained with Hoechst 33342 (1:2,000; H3570, Life Technologies) or DAPI (0.5 µg/mL; 10236276001, Roche).

Confocal microscopy images, shown as maximum projected Z-stack images, were acquired with a Leica SP5 laser scanning confocal microscope and LAS AF Lite software (Leica Microsystems). Images were taken using 40X oil submerged objective and analysed using ImageJ, p65+ cells were quantified by manually counting 9 images per condition from four patient lines. Images of infected cells were acquired with EVOS™ FL Digital Inverted Fluorescence Microscope (Invitrogen), using 10X or 20X objective.

Supplementary tables

Table S1. Donor list for this study.

Organoid type	Patient #	Gender	Age	Surgery
Jejunum	44	F	34	Gastric by-pass
Jejunum	48	M	50	Gastric by-pass
Jejunum	50	M	53	Gastric by-pass
Jejunum	84	F	44	Gastric by-pass
Jejunum	119	F	49	Renewal of the gastrojejunostomy because of an ulcer

Table S2. List of bacterial strains used in this study.

EPEC derivative	Genotype	Description	Source
E2348/69	wt, O127:H7	Added GFP plasmid	Litvak et al., 2017
SS6108	*ΔnleB*, *ΔnleE*, *Δnlec*, *ΔnleD*	derived from strain E2348/69, O127:H7, added GFP	Litvak et al., 2017
